# Therapeutic Innovations in Nasopharyngeal Carcinoma: Current Strategies and Emerging Perspectives

**DOI:** 10.3390/life16050764

**Published:** 2026-05-02

**Authors:** Weronika Pająk, Jakub Kleinrok, Joanna Pec, Adrian Orzechowski, Jakub Drabko, Ryszard Sitarz, Alicja Forma, Adam Brachet, Barbara Teresińska, Jacek Baj

**Affiliations:** 1 Department of Forensic Medicine, Medical University of Lublin, Jaczewskiego 8b, 20-090 Lublin, Poland; apec1410@gmail.com (J.P.);; 2 Chair and Department of Clinical Pathomorphology, Medical University of Lublin, Jaczewskiego 8b, 20-090 Lublin, Poland; klejs.90@gmail.com; 3 1st Department of Psychiatry, Psychotherapy and Early Intervention, Medical University of Lublin, Gluska Street 1, 20-439 Lublin, Poland; 4 Department of Correct, Clinical, and Imaging Anatomy, Medical University of Lublin, Jaczewskiego 4, 20-090 Lublin, Poland; adambrachet@gmail.com (A.B.);; 5 Doctoral School, Medical University of Lublin, Chodźki 7, 20-093 Lublin, Poland; jacek.baj@umlub.edu.pl

**Keywords:** therapeutic strategies, nasopharyngeal carcinoma, immunotherapy, targeted therapy, radiotherapy, precision medicine

## Abstract

Nasopharyngeal carcinoma (NPC) presents unique clinical and biological characteristics that distinguish it from other head and neck malignancies. It poses a great therapeutic challenge for many specialists. It is associated with Epstein–Barr virus (EBV) infection, genetic predisposition, and environmental risk factors. With advancements in radiotherapy and systemic therapy, new treatment options have emerged. We want to focus on contemporary therapeutic strategies for NPC, emphasizing breakthroughs in intensity-modulated radiotherapy (IMRT), chemoradiotherapy, targeted therapy, immunotherapy, and emerging cellular therapies. By integrating recent discoveries with clinical evidence, we aim to provide state-of-the-art information, along with a comprehensive understanding of current best practices, emerging treatments, and critical prognostic determinants in NPC.

## 1. Introduction

Nasopharyngeal carcinoma (NPC) is one of the most outstanding malignant cancers, originating from the mucosal epithelium of the nasopharynx. It is biologically, clinically, and epidemiologically distinct from other head and neck squamous cell carcinomas (HNSCC) [1]. It is linked to the Epstein–Barr virus (EBV) infection, genetic predispositions, and an overall tendency for early regional lymphatic spread and distant metastasis [2].

NPC is a very common malignancy with an extreme geographical distribution. It occurs rarely in several countries (mainly European/North American regions), but is highly endemic in areas including Southeast Asia, southern China, and northern Africa [3,4]. Recent global epidemiological projections indicate that NPC incidence and mortality rates will increase sharply by 2040. The disease burden is growing and is sex-associated; further, it is inversely correlated with the Human Development Index, indicating the importance of ongoing optimization of the treatment paradigm across the globe [3,4].

From an anatomical perspective, the nasopharynx lies at the very base of the skull and close to critical structures, including the brainstem, spinal cord, and cranial nerves. As a result of this complex anatomical composition, primary surgical resection is technically difficult and associated with a high prevalence of serious complications, which preclude its potential as a first-line treatment [1].

Nowadays, the traditional open surgical approach, like the maxillary swing, is mostly being replaced by minimally invasive approaches, with endoscopic endonasal nasopharyngectomy being the primary one [5]. The Draf III procedure is an endoscopic technique that could be utilized for the treatment of NPC tumors that are located in the skull base and, in the case of a paranasal tumor with high success rate for both primary (83.5%) and revision (71%), pairing with the usage of mucosal flaps instead of stents can lead to the improved efficacy of the procedure (87% vs. 72%) [6]. Although modern endoscopic methods have shown much lower morbidity and improved visualization than in earlier decades, surgery is considered salvage only for selected patients who exhibit localized recurrences after definitive irradiation [1,5]. As the foundational idea of the treatment for NPC is non-operative treatment, we have purposely omitted surgical guidelines from the review, summarizing knowledge on modern radiotherapy and systemic drug therapies, non-surgical strategies of primary NPC care [7,8]. The use of intensity-modulated radiotherapy (IMRT) is revolutionizing NPC treatment, providing outstanding locoregional control with spared critical adjacent normal healthy tissues [9,10]. In locoregionally advanced disease, IMRT is a standard treatment with concurrent platinum-based chemotherapy [7]. Although these are advantageous clinical outcomes, distant metastasis is still the most prevalent mechanism underlying treatment failure and the leading cause of death [10,11]. In the past few years, a paradigmatic shift brought about by systemic therapies to address these persistent hurdles has been underway. Meanwhile, targeted therapies and immunotherapy, specifically programmed death-1 (PD-1) blockers, were shown to be highly efficacious with respect to improving the survival outcome [12].

Therefore, the goal of this review is to provide a detailed overview of new techniques outside surgery for NPC as well as to combine cutting edge evidence with clinical findings to illustrate best practices, persistent challenges, and main prognostic elements in NPC. Moreover, we aim to comprehensively analyze contemporary NPC treatment strategies, covering clinical and biological aspects of each therapy. An emphasis has been put on biomarkers and precision medicine, which are key factors shaping the future treatment.

## 2. Methods

This article was designed as a narrative review. A targeted literature search was performed in PubMed, Scopus, and Web of Science for articles published between January 2010 and April 2026, with the final search update performed in April 2026. This time frame was selected to focus on contemporary therapeutic strategies, including modern intensity-modulated radiotherapy, systemic therapy optimization, immunotherapy, and biomarker-driven approaches. The search strategy used combinations of the following keywords: “nasopharyngeal carcinoma”, “radiotherapy”, “IMRT”, “chemoradiotherapy”, “induction chemotherapy”, “immunotherapy”, “targeted therapy”, “biomarkers”, “precision medicine”, and “survival”.

The study selection process was performed in a structured but non-systematic manner, consistent with the narrative character of the review. First, titles and abstracts were screened for relevance to contemporary non-surgical management of nasopharyngeal carcinoma. Second, potentially relevant full-text articles were assessed, with priority given to clinical trials, meta-analyses, observational studies, and guideline-based papers. Selected preclinical studies were included when necessary to explain biological mechanisms or emerging therapeutic directions. Additional relevant articles were identified through manual screening of reference lists of key publications.

Publications were included if they addressed radiotherapy, chemoradiotherapy, systemic therapy, immunotherapy, targeted therapy, biomarkers, or precision medicine in nasopharyngeal carcinoma. Studies primarily focused on surgical management, non-nasopharyngeal head and neck cancers without separate nasopharyngeal carcinoma-specific data, or topics outside the scope of contemporary therapeutic strategies were excluded. Because this was a narrative review, the evidence was synthesized qualitatively, and no formal systematic review protocol, PRISMA-based study selection procedure, or risk-of-bias assessment was performed.

## 3. Risk Factors

The pathogenesis of NPC is multifactorial and complex. In contrast to the majority of the other head and neck cancer-related diseases, which are a result of tobacco and alcohol use, NPC develops as a complex interaction of viral infection, genetic vulnerability, and environmental exposure [13,14]. We have separated risk factors into non-modifiable and modifiable determinants to give a clearer picture.

### 3.1. Non-Modifiable Risk Factors

#### 3.1.1. EBV Infection

EBV infection is the most well-established and critical non-modifiable risk factor for endemic NPC. While EBV infects over 90% of the global population, it typically remains latent [15]. However, in susceptible individuals, the virus establishes a persistent latent infection in the nasopharyngeal epithelial cells. The expression of latent viral proteins (such as LMP1 and LMP2) and non-coding RNAs plays a pivotal role in driving malignant transformation by promoting cell proliferation, inhibiting apoptosis, and evading immune surveillance [16,17]. The quantification of circulating plasma EBV DNA has even become a primary biomarker for NPC screening and prognostication [18].

#### 3.1.2. Genetics

The geographical distribution of NPC strongly indicates its significant genetic tendency. A large proportion of this risk is linked to certain Human Leukocyte Antigen (HLA) class I genes (e.g., HLA-A*02:07) that block the immune system’s mechanism to eliminate EBV-infected cells [19,20]. In addition, patients with a first-degree relative with NPC are at significantly increased risk for developing NPC, emphasizing the role of familial clustering [21]. Analyses of both age and sex have also shown that incidence peaks at 40 to 60 years of age, with males being roughly two to three times as likely to develop NPC [22].

### 3.2. Modifiable and Environmental Risk Factors

#### 3.2.1. Diet

Diet plays a crucial part in the cause of NPC, especially in endemic areas. The eating of traditional salt-cured foods, such as Cantonese-style salted fish and preserved vegetables, is associated with the disease [23]. These contain high quantities of highly volatile nitrosamines, which are highly toxic. The chronic, early childhood, or weaning exposure to such dietary carcinogens increases the lifelong risk of NPC [14,24]. On the other hand, diets containing lots of fresh fruits and vegetables with antioxidants exert a protective effect [25].

#### 3.2.2. Smoking and Drinking

Although tobacco and alcohol are the main risk factors for other HNSCC, they are not as prominent in NPC. Cigarette smoking is closely related to NPC risk, particularly in differentiated squamous cell subtypes, and can act synergistically with EBV infection in promoting tumor development [26,27]. Heavy drinking has come to stand as an independent risk factor in contributing to general mucosal susceptibility to carcinogenic insults [28].

#### 3.2.3. Occupational Exposure

Excessive exposure in the workplace to a range of inhaled irritants and chemicals is another major modifiable risk factor. Persistent inhalation of wood dust, formaldehyde, and chemical fumes (commonly observed in the carpentry, textile, and chemical industries) has been demonstrated to promote chronic inflammation in the nasopharyngeal mucosa, aiding epithelial dysplasia and resulting in malignant development [29].

**Figure 1:**
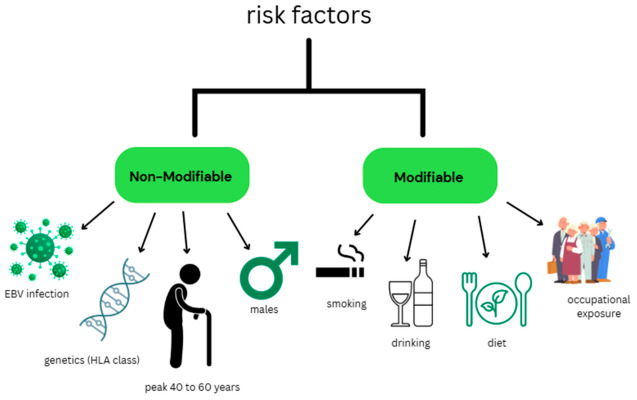
Risk factors of nasopharyngeal carcinoma.

Risk factors of nasopharyngeal carcinoma are shown in Figure 1.

## 4. Pathology of Nasopharyngeal Carcinoma

NPC, also known as lymphoepithelioma or lymphoepithelial carcinoma in the past, is a malignant neoplasm developing from mucosal epithelium in the nasopharynx [30]. The current WHO classification (2022) distinguishes three morphologic types of NPC: (i) keratinizing squamous cell carcinoma (SCC) (K-NPC), (ii) nonkeratinizing SCC (NK-NPC), and (iii) basaloid SCC [31]. From the histologic point of view, K-NPC resembles conventional keratinizing squamous cell carcinoma, whereas NK-NPC, particularly in its undifferentiated form, is more akin to poorly differentiated carcinoma with a syncytial growth pattern and prominent lymphoid stroma [30,31,32]. K-NPC is characterized by evident squamous differentiation with keratin pearl formation and intercellular bridges, thus resembling conventional keratinizing squamous cell carcinoma of other head and neck locations. Nonkeratinizing NPC, by contrast, typically consists of syncytial sheets, nests, or trabeculae of malignant epithelial cells with indistinct cytoplasmic borders, enlarged vesicular nuclei, and prominent nucleoli, usually accompanied by a dense lymphoid-rich stromal background. This subtype may be further appreciated as differentiated or undifferentiated, depending on the degree of recognizable squamous maturation [32,33,34]. Basaloid SCC is a rare subtype that histologically resembles basaloid squamous cell carcinoma arising at other head and neck sites [31]. It is composed of compact nests and lobules of small to medium-sized basaloid cells with hyperchromatic nuclei, scant cytoplasm, high mitotic activity, and frequently, central comedo-type necrosis [33,34]. NK-NPC, especially in endemic regions, is strongly associated with EBV, and EBER in situ hybridization is commonly used as an ancillary diagnostic tool [30]. Immunohistochemical analysis of NPC usually shows epithelial differentiation with positivity for pan-cytokeratins, while squamous differentiation may be highlighted by p40 and p63 [31].

NPC staging currently uses the American Joint Committee on Cancer/International Union Against Cancer (AJCC/UICC) version 9 TNM classification, which was published in 2025. In this system, T1 indicates disease limited to the nasopharynx or extending to the oropharynx or nasal cavity without affecting the parapharyngeal space. T2 includes parapharyngeal or adjacent soft-tissue extension. T3 refers to invasion of the bony skull base, cervical vertebrae, pterygoid muscles, or paranasal sinuses. T4 includes intracranial, cranial nerve, orbital, parotid, hypopharyngeal, or extensive soft-tissue extension. Nodal staging considers laterality, size, nodal level, and radiologic extranodal extension. Metastatic disease is divided into M1a and M1b categories, based on the extent of metastasis [35,36].

## 5. Advances in Radiotherapy for Nasopharyngeal Carcinoma

### 5.1. Precision Radiotherapy Techniques

#### 5.1.1. Proton Therapy

Advantages of proton therapy (PRT), compared to traditional photon radiotherapy, include a sharp dose fall off, which decreases damage to surrounding tissues and allows for dose escalation in the tumor area. Compared to intensity modulated radiotherapy, PRT spares normal tissue while maintaining effective delivery of the dose to the targeted areas [37,38,39]. Comparing three-dimensional conformal radiation with IMRT and PRT verified that patients treated with PRT had the best dose coverage with respect to PTV area, while maintaining a better protection of the spinal cord and parotid glands [40]. Researchers from Massachusetts General Hospital presented their experience in PRT for locoregionally advanced nasopharyngeal carcinoma (LANPC) patients. Of the 19 patients with a T4 stage involved in the study’s 3-year overall survival (OS) rate, progression-free survival (PFS) and locoregional free survival rates were 74%, 75%, and 92%, respectively [41]. In the phase II study launched by the MGH, they evaluated the effect of combination proton–photon therapy (70 Gy in 35 daily fractions) combined with cisplatin and fluorouracil. On follow-up, after 28 months, the local control rate was 100%, with the same for 2-year OS, and the disease-free survival rate was 90%. The biggest toxic effect was the need to place gastrostomy tubes for half the patients. Other toxic effects included hearing loss (29%), and weight loss (38%). There were also no grade-3 xerostomia cases reported [42]. The effectiveness of proton therapy gives promising data; however, it still needs to be further studied. The dosimetric superiority of proton therapy was achieved thanks to pencil-beam scanning and IMPT, which both can be further developed, including in vivo verification prior to clinical implementation. The second issue is the small number of cancer centers (128 in the world, with most being located in USA, Japan, Europe and China) with ways of delivering proton therapy, as well as the high cost that these therapies entail, thus severely limiting access to this type of therapy for many patients [43,44]. There has also been only small clinical studies on this treatment method, and a clinical study with a large number is still needed to confirm the best treatment combination and efficacy of PRT.

#### 5.1.2. Adaptive Radiotherapy

Adaptive radiotherapy (ART) is a relatively new concept of radiotherapy; ART means replanning of the target design during treatment. The concept is based on the fact that patients’ anatomies will change throughout radiotherapy. There are reports that target volume shrinkage and weight loss are quite common for NPC patients undergoing radiotherapy [45]. ART means to address the impact of these changes on the planned dose distribution during treatment. The volume and OARs are commonly at the limit of their prescribed tolerances during NPC radiotherapy, and replanning can be essential to ensuring the dosage remains within tolerance and avoids unnecessary damage to patients’ organs [46]. Comparing the survival outcomes of patients with and without adaptive replanning shows that for patients treated with ART, the 2-year locoregional relapse-free survival was 88%, while for the patients undergoing standard radiotherapy treatments, that metric amounted to 79%. Identifying patients who would benefit from ART might be a challenge, and it is difficult to determine clear guidelines on this issue. Data from available studies has shown that only around 5% of patients needed replanning for protection of the brachial plexus. Dose distribution was actually greater than planned doses; however, the dose increase was around 1% [47,48,49]. The organ that benefits the most from using ART is the parotid gland, as it has the greatest volume decrease during treatment (26% average), and damage to the gland may often lead to xerostomia, which may greatly impact the patients’ quality of life. While traditional offline ART (using weekly cone-beam CT or CBCT) is widely accessible, online ART (real-time adaptive planning on the treatment machine) is emerging, particularly in advanced centers, using CT-linac or MR-linac systems, which may cause limitations with the wider adoption of online ART in the treatment of NPC [50]. Due to heterogeneity, there are no clear guidelines on when the ART is beneficial, and it needs to be analyzed on a case-by-case basis to get the best treatment outcome.

### 5.2. Radiotherapy-Based Combination Approaches

#### 5.2.1. Concurrent Chemoradiotherapy

Concurrent chemoradiotherapy (CCRT) is a combination technique that combines the usual radiotherapy with chemotherapy and is useful in the treatment of locally advanced cases of NPC. The primary chemotherapy relies on platinum-based agents, which can create cross bindings in DNA structure, blocking the growth of the cells, which ultimately leads to apoptosis-mediated tumor shrinkage. According to studies, the combined approach increased the patients’ PFS from 24% to 69%, and the 3-year survival rate went from 47% to 78% [51]. These results show that combining radiotherapy with chemotherapy yields much better results than radiotherapy alone, and other later studies only reaffirmed these results [52,53,54,55,56,57]. Another study, where CCRT was combined with ICT and ACT, showed that, while not giving a lot of short-term benefits for overall survival for patient groups with (80%) and without (76%) ICT in a 5-year scope, extending the observation to 90 months showed that including IC in the group yielded better treatment outlooks with an OS of 76% compared to 70% for patients without IC, and PFS of 76% vs. 71%, and the addition of adjuvant chemotherapy helped significantly, with a 5-year OS of 82% compared to 72% for groups without ACT, and, similarly, PFS was 81% and 69% for groups with ACT and without, respectively. This suggests that CCRT, in combination, can be an effective treatment method with significant potential for integration with other therapeutic approaches, offering patients a better overall chance for both OS and PFS while maintaining their QOL to a greater degree [58].

All things considered, CCRT has benefited the treatment of NPC by greatly increasing patients’ survival rates and becoming one of the fundamental treatments in locally advanced NPC.

#### 5.2.2. Immunotherapy

NPC shows suitability for immunotherapy since studies have shown that the cancer cells show high expression of EBV antigens and CD4^+^/CD8^+^ T-cell target proteins [59,60], and expression of PD-L1 of up to 89–95% [61]. However, due to an immunosuppressive tumor microenvironment (TME), NPC can proliferate.

Based on current knowledge, the best approach to immunotherapy for NPC would be using Immune Checkpoint Inhibitors (ICIs). Combining immunotherapy with radiotherapy may utilize the radiation to recruit tumor-associated macrophages and cause an upregulation of PD-L1 that could give a good opportunity for PD-L1 blocking therapies to work [62]. Most of the available studies have focused on exploring the safety of combining these two approaches. The closest results we have are from a phase II trial which investigated the effect of neoadjuvant pembrolizumab followed by surgery, then supplemented with adjuvant pembrolizumab-RT or pembrolizumab-cisplatin-RT, in HNSCC patients. At the interim analysis, none of the 16 patients in the pembrolizumab-RT arm had grade 4 toxicities due to dose-limiting toxicities, which led them to conclude that combining PD-1 inhibitors with radiotherapy is safe for HNSCC [63], which could lead to exploring if the same were true for NPC. In a multi-center study that looked into patients with NPC with distant metastasis, with many having at least two metastasis sites upon treatment administration of PD-L1 inhibitors, within a minimum treatment time of 6 months, patients with non-liver metastasis had significantly longer PFS of 144 days vs. 72 days and OS of 730 days vs. 305 days. However, lung, bone, and lymph-node metastasis had no significant difference in PFS and OS. The study also showed that patients with liver metastasis and fewer other metastatic sites had shorter OS compared to patients with a higher count of metastasis overall without metastatic sites in the liver, concluding that patients with liver metastasis have poor survival patterns when receiving anti-PD-L1 treatment [64]. A phase 3 clinical trial for sintilimab (pd-1 inhibitor) which focused on patients with LANPC, with the exclusion of patients with T3-4N0 and T3N1 stages of disease, showed that including PD-1 inhibitors in the treatment plan raised 3-year event-free survival from 76% to 86%; however, 74% of patients receiving sintilimab developed grade 3–4 adverse effects compared to 65% for the group receiving standard treatment. Having two (1%) patients die in the sinitilmab group (both considered to be immune-related) with one person dying in the standard treatment group, these results lead to the conclusion that, while the addition of sintilimab in the treatment of LANPC is beneficial for improving PFS, it leads to higher but manageable adverse effects, and a longer follow-up is necessary to conclude whether this treatment could be a standard of care for patients with high-risk LANPC [65]. Currently, there are ongoing clinical trials into the efficacy of combining immunotherapy with chemotherapy and radiation in the treatment of NPC, but there are no large-scale conclusive results available at this moment.

## 6. Systemic Therapy Innovations

### 6.1. Chemotherapy Developments

Chemotherapy remains the main form of treatment for NPC, as most of the NPC incidences are chemosensitive. This form of treatment is especially helpful in locoregionally advanced, metastatic, or recurrent incidences. In the past years, chemotherapy treatments have undergone optimalization of induction chemotherapy (ICT) and their integration with other treatment methods, which led to better OS and PFS [66].

There are two main ICTs which combine docetaxel, cisplatin, 5-fluorouracil (TPF) and platinum-gemcitabine (GP) [67]. They have been widely compared and analyzed in multiple studies, with the results of comparable OS and PFS between the regimens. The choice of ICT depends on the regimen toxicity and patients’ profile, with higher toxicity reported with TPF [68,69]. In the cases of LANPC, it is advised to provide two cycles of ICT as more cycles do not improve OS or PFS but lead to a higher incidence of acute toxicities [70].

A crucial direction of research is faced toward combined treatments in NPC, especially CCRT. Studies report that providing ICT before CCRT may lead to better disease control. Moreover, combining those treatments has shown higher OS and PFS [71,72,73,74].

Moreover, the modern role of ICT in the NPC treatment should be considered in the broader context of constantly changing therapeutic paradigms. The newest studies highlight that ICT is a crucial element of LANPC treatment strategies, especially when combined with CCRT, and more often with immunotherapy [75]. Randomized studies show that providing ICT before CCRT leads to significantly higher PFS and OS with better metastasis control [76,77]. Furthermore, including ICIs into ICT-based treatment paradigms shows promising results, suggesting further evolution of treatment standards toward multimodal treatments with high efficiency and acceptable safety profiles [78].

In the past years, particular attention was brought to other methods of NPC treatment. Apart from the standard regimen of ICT and CCRT, combining ICT with immunotherapy emerged. Clinical trial results indicate that adding ICIs to standard chemotherapy regimens leads to significant improvement in treatment outcomes. What is more, by adding PD-1 inhibitors to TPF or GP regimens, better OS and PFS are observed. Thereby, chemotherapy is no longer a monotherapy but a part of multimodal therapy [79,80].

### 6.2. Targeted Therapies

The development of targeted therapies for NPC focuses mainly on the identification of particular molecular mechanisms behind the pathogenesis of NPC, especially connected with EBV. In contrast to other HNSCC, NPC characterizes a distinct molecular profile, which makes it difficult to identify a single dominant therapeutic target [81,82]. HNSCC are linked to smoking, high genetic mutations burden, and oncogenic drivers, whereas NPC is strictly connected to EBV infection and shows a relatively low number of genetic mutations [83,84]. Viral factors, epigenetic changes, and interactions in the TME play a key role in NPC pathogenesis. Such characteristics limit the efficiency of therapies targeted at particular signaling pathways, as there is no dominant molecular aim. Moreover, these actions may lead to the activation of alternative pathways and, furthermore, cause resistance mechanisms. It helps one to understand why multimodal therapies, especially immunotherapy combined with chemotherapy or radiotherapy, show better clinical effects than targeted therapies [85,86,87].

The EGFR pathway is one of the most frequently studied; however, studies on EGFR inhibitors in NPC remain inconclusive. At the same time, anti-angiogenic therapies, which target the VEGF pathway, have shown antitumor activity. Unfortunately, their clinical role has not yet been clearly established [88,89].

### 6.3. Immunotherapy in Nasopharyngeal Carcinoma

In recent years, immunotherapy has shown its great potential in the treatment of NPC, changing the traditional therapeutic approach. It can be divided into two main groups: active and passive immunotherapy. The first one includes ICIs, vaccines, and immunomodulators. The latter is based on adoptive cell transfer (ACT) and EBV-specific monoclonal antibodies [90]. ICIs are especially important in the treatment of recurrent and metastatic NPCs. Combined with ICT, they enhance OS and PFS. Particularly promising are the anti-PD-1 and anti-PD-L1 antibodies. Randomized trials have reported that adding PD-1 inhibitors to traditional chemotherapy improves survival outcomes [91,92]. Recently, three phase 3 trials were conducted: JUPITER-02, CAPTAIN-1st, and RATIONALE-309. They tested toripalimab with first-line chemotherapy in patients with recurrent or metastatic NPC. The studies showed a significantly higher OS and PFS. In the JUPITER-02 trial, the use of toripalimab in combination with GP led to a significant prolongation of PFS, compared to chemotherapy alone. Similar results were obtained in the CAPTAIN-1st (camrelizumab + GP) and RATIONALE-309 (tislelizumab + chemotherapy) studies, confirming the efficacy of the combination therapy strategy. Additionally, treatment with toripalimab was associated with favorable changes in EBV DNA levels, which may serve as a marker of disease progression, and the safety profile of the therapy was similar to that of chemotherapy alone. Consequently, combination immunotherapy has become the new standard for first-line treatments for patients with NPC [12,93,94,95,96].

A modern, widely researched form of treatment for NPC is re-challenging with immunotherapy after experiencing disease progression, following the treatment with PD-1 inhibitors. At the same time, adjuvant treatment strategies are being developed, in which PD-1 inhibitors follow CCRT for further improvement of disease control [95,97,98,99].

One of the biggest challenges facing today’s NPC treatment is finding a reliable predictive biomarker for immune response to immunotherapy. PD-L1 expression is the most studied marker. However, studies about its significance as a prognostic biomarker in NPC remain inconclusive. Of increasing importance are EBV DNA levels, TME characteristics, and the presence of tertiary lymphoid structures. Nevertheless, more studies should be conducted [97,100,101].

One of the directions of treatment research was directed towards EBV infection and its effects on NPC. Emerging therapeutic vaccines show a promising treatment strategy, as the correlation between the NPC occurrence and EBV infection was proven. Viral proteins, such as LMP1 and BRLF1, may regulate PD-L1 expression and contribute to the immune evasion of cancer cells [102,103]. Various vaccine approaches are studied, including vaccines based on dendritic cells, viral vectors, peptides, DNA, and modern mRNA platforms. Although results demonstrate safety and the ability to activate the immune system, clinical efficacy remains limited and requires further study [104,105].

Cell therapies that have emerged in recent years are clinically vital, including the adoptive transfer of EBV-specific T cells. A clinical trial by Louis et al. (2010) [106] has reported that this approach may lead to durable responses in refractory cases [106]. Modern CAR-T technology enables the genetic modification of T cells, therefore enhancing their ability to recognize cancer cells expressing EBV antigens [88]. Additionally, research on NK cells shows their potential role in eliminating EBV-infected cells, which could be used in combined therapy [107].

Nevertheless, immunotherapy for NPC still faces multiple challenges. The most important ones are the treatment resistance, the lack of standardized predictive biomarkers and the complex interactions between the tumor and the immune system. Therefore, emphasis should be put on overcoming present hindrances to improve NPC treatment outcomes.

## 7. Biomarkers and Precision Medicine

### 7.1. Epstein–Barr Virus DNA

One of the best-described tumor markers for NPC is circulating free EBV DNA. Infection with this virus appears to play a significant role in the pathogenesis of NPC, and the EBV genome can be detected in many NPC tumors [108]. The high incidence of NPC in Southeast Asian countries is often associated with the high incidence of EBV infection in this region [109]. Due to the existing correlation between EBV and NPC, the potential use of EBV-free DNA in early detection and monitoring of disease progression and response to treatment is being discussed [108]. It has also been observed that there is a correlation between EBV DNA levels and patient prognosis at various time points during treatment [109].

Kim et al. described EBV viral DNA as a useful marker for monitoring treatment responses and for post-treatment surveillance. They emphasize its potential for detecting disease recurrence several months earlier than standard methods. However, in order for the EBV DNA test to be therapeutically relevant in this situation, it is necessary to identify the timing and frequency of testing following treatment completion. Additionally, temporary elevations during treatment, as well as potential interpretation errors, should be considered [110].

Neo et al. demonstrated the dynamic variability of plasma Epstein–Barr virus (pEBV) DNA levels over time following radiotherapy, thereby emphasizing the importance of timing sample collection from patients. EBV DNA detected 0–2 weeks after radiotherapy was associated with poorer PFS, but samples collected 8–12 weeks after treatment provided a more accurate assessment of the risk of recurrence [111]. In another study, Neo et al. also demonstrated the possibility of using EBV DNA to monitor patients after completing radiotherapy for potential recurrence. Negative results had a high negative predictive value, while the positive predictive value was moderate and depended on the threshold. A copy number greater than 500 per milliliter was considered to indicate a higher risk of recurrence [112].

In a study by Lertbutsayanukul et al., the presence of detectable EBV DNA after treatment (post-EBV) in patients who underwent IMRT alongside chemotherapy was identified as a significant adverse prognostic factor. This was independently associated with poor OS, PFS, and distant metastasis-free survival (DMFS). Furthermore, EBV DNA detected at week 5 of radiotherapy (mid-EBV) was associated with poorer OS and PFS [113].

Ma et al. described the possibility of using pEBV DNA clearance assessment alongside PET-CT response four weeks after treatment as dual endpoints. This would have prognostic (OS and PFS) and predictive significance and may be a tool for assessing early response to treatment [114].

Pramanik et al. conducted a study in a region where NPC is not endemic. pEBV DNA was detected in most patients, with an increased number of copies per milliliter observed as the disease progressed. A decrease in viral load was also observed following both induction and definitive chemotherapy. However, in the event of disease recurrence, there was a sharp increase in EBV copy number [115]. Furthermore, serological detection of elevated EBV DNA levels in patients can be used as a complementary screening test for NPC. Chen et al. emphasized in their prospective cohort study that elevated EBV DNA levels may correlate with the risk of developing NPC within 3 years. However, the authors stress the need for further research, particularly to assess patterns of fluctuation in EBV DNA levels over time [116].

EBV DNA can also be used to indicate when it is safe to reduce the intensity of therapy for some patients. Lee et al. emphasized the potential of using EBV DNA levels alongside imaging techniques to identify patients who could benefit from this approach [117]. In summary, EBV DNA currently appears to be one of the most clinically useful biomarkers in NPC. Its broader application is limited by methodological variability, the lack of standardized cutoff values, and reduced test sensitivity in certain clinical situations, such as the early stages of the disease or small-volume local recurrence [118].

### 7.2. Genomic, Transcriptomic, and Liquid Biopsy Markers

Precision diagnostics offer additional opportunities for the early detection of NPC and may support prognosis assessment and treatment stratification. The most important current areas include mutation analysis, transcriptomic profiling, liquid biopsy, and DNA methylation [119]. Genomic and transcriptomic analyses may help identify biological pathways involved in NPC development and progression and may support future risk stratification by integrating molecular features with clinical characteristics [120]. Transcriptomic profiling may provide insight into TME heterogeneity, including immune-related and cellular signatures associated with tertiary lymphoid structures. This may be relevant for prognosis and response to immunotherapy [121,122,123]. Multi-marker signatures may also better reflect tumor complexity than individual biomarkers and may improve patient stratification, particularly in the context of immune checkpoint blockade [124].

Liquid biopsy can complement tissue profiling by enabling repeated, minimally invasive sampling and potentially better capture of tumor heterogeneity over time [125,126]. In addition to EBV-based assays, ctDNA, cfDNA, miRNAs, and DNA hypermethylation are potentially helpful methods with prospective uses in early diagnosis, treatment–response evaluation, minimal residual disease detection, and disease monitoring [126,127,128,129,130]. Some emerging transcriptomic tools, including RNA-seq-based classifiers, may also help identify patients more likely to benefit from immunotherapy in the future [131].

However, despite their potential, these methods are still limited by early clinical data, a lack of prospective validation, different methods, and a lack of standardization. Therefore, they should currently be regarded as promising translational tools rather than established biomarkers for routine clinical practice [120,121,122,123,124,125,126,127,128,129,130,131].

### 7.3. Imaging Biomarkers

Imaging methods such as CT, MRI, and PET/CT currently form the basis of NPC diagnosis and treatment planning. Furthermore, imaging has become an increasingly important source of quantitative markers in recent years [132,133].

Important areas of development include radiomics, deep learning models, and AI. These approaches can convert routine imaging data into quantitative features that may support prognosis prediction, response assessment, and treatment stratification, particularly when combined with clinical variables such as stage and plasma EBV DNA [134,135,136,137]. MRI- and PET/CT-based models appear especially promising, as they may improve prognostic evaluation and provide additional value beyond conventional imaging alone [135,136,137].

**Figure 2:**
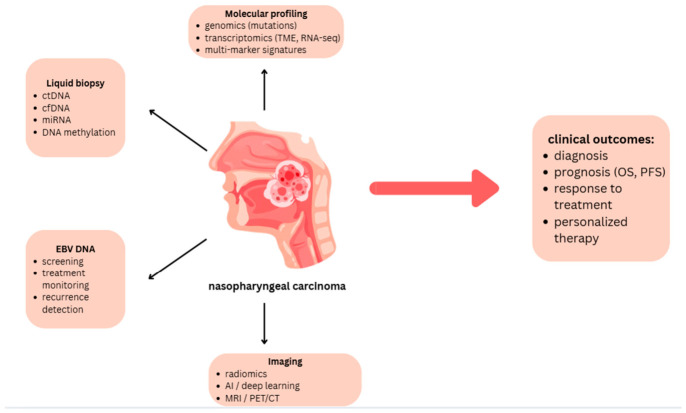
Biomarkers and precision medicine in NPC.

AI and deep learning can be used for both diagnosing and planning the treatment, including image interpretation, automatic segmentation, and workflow optimization [138,139,140]. However, it should be emphasized that the currently available studies are characterized by considerable heterogeneity and a risk of publication bias. This is why further validation and standardization of methods is important before they can be applied more widely [139]. In particular, large-scale multi-center external validation using independent datasets is necessary to improve the reproducibility, reliability, and generalizability of these models [141,142]. The standardization of radiomic processes and image capture is equally crucial because the absence of consistent protocols significantly restricts clinical translation and cross-study comparison [143,144]. Biomarkers and precision medicine methods in NPC are shown in Figure 2.

## 8. Discussion

NPC remains a biologically and clinically distinct malignancy among head and neck cancers. Therapeutic progress in this disease has resulted primarily from improvements in non-precision treatment modalities. As outlined in our review, the introduction of IMRT fundamentally changed the standard of care in NPC, enabling excellent locoregional control while simultaneously sparing normal tissues and critical structures [7,8,9,10,11]. In patients with locally advanced disease, the cornerstone of treatment remains the combination of IMRT with concurrent platinum-based chemotherapy, confirming that effective NPC therapy still requires the integration of high-quality local and systemic treatment [7,51,52,53,54,55,56,57,58,59]. However, it is important to note that, despite improved local outcomes, distant metastases remain the most frequent mechanism of treatment failure and the leading cause of death, clearly demonstrating that further survival improvement is unlikely to be achieved solely through intensification of local therapy [10,11]. However, these advances should be interpreted with caution, as better locoregional control has not eliminated the persistent problem of distant metastasis. This suggests that technical improvements in local treatment, although essential, may not be sufficient to substantially improve long-term survival without more effective systemic strategies [7,8,9,10,11]. It also highlights an important gap in the current literature, namely, the limited availability of studies directly comparing how improvements in local control translate into overall survival across different clinical settings [7,8,9,10,11].

Another important area of development includes more advanced radiotherapeutic techniques, such as proton therapy and adaptive radiotherapy. Proton therapy offers dosimetric advantages over conventional photon radiotherapy, allowing better sparing of critical organs while maintaining adequate target volume coverage [37,38,39,40,41,42]. Adaptive radiotherapy, in turn, addresses the dynamic anatomical changes that occur during treatment, such as weight loss and reductions in tumor and salivary gland volume, which may translate into better agreement between the actually delivered dose and the original treatment plan [45,46,47,48,49]. At the same time, it must be stressed that both approaches still face important practical limitations. In the case of proton therapy, major challenges include limited availability and the lack of large prospective studies, whereas for adaptive radiotherapy, clear criteria for selecting the patients who are most likely to benefit have not yet been established [37,38,39,40,41,42,43,44,45,46,47,48,49]. For these reasons, these strategies should currently be regarded as promising components of future therapeutic optimization rather than as universally established solutions across all patient groups. Importantly, the current evidence supporting proton therapy and adaptive radiotherapy remains stronger at the dosimetric and technical level than at the level of robust comparative survival data [37,38,39,40,41,42,43,44,45,46,47,48,49]. In particular, proton therapy is limited by restricted availability, cost, and the paucity of large prospective studies, whereas adaptive radiotherapy still lacks standardized criteria for patient selection, timing of replanning, and clinically validated thresholds for benefit [37,38,39,40,41,42,43,44,45,46,47,48,49]. Thus, these strategies appear promising, but their precise place in routine NPC management remains incompletely defined [37,38,39,40,41,42,43,44,45,46,47,48,49].

An even more important development in contemporary NPC management is the progress in systemic therapies. Induction chemotherapy remains an important component of treatment for patients with LANPC, with the TPF and GP regimens appearing to provide comparable outcomes, although they differ in toxicity profiles [66,67,68,69,70,71,72,73,74]. Particularly noteworthy is the fact that, in recent years, chemotherapy has ceased to be viewed as an isolated treatment modality and has increasingly become part of multimodal strategies that also include immunotherapy [79,80]. The most practice-changing data come from phase III trials such as JUPITER-02, CAPTAIN-1st, and RATIONALE-309, which demonstrated that adding PD-1 inhibitors to chemotherapy in the first-line treatment of recurrent or metastatic NPC improves both overall survival and progression-free survival [93,94,95,96]. These results are biologically plausible, given that the pathobiology of this malignancy is closely linked to EBV infection, high expression of viral antigens, frequent PD-L1 expression, and an immunologically active yet functionally immunosuppressive TME [59,60,61,62]. Nevertheless, it should be emphasized that data regarding the combination of immunotherapy with radiotherapy remain limited, and part of the rationale is still extrapolated from results obtained in other head and neck cancers rather than directly in NPC [62,64]. For this reason, this approach appears highly promising, but it still requires further clinical investigation. At the same time, the strength of evidence is not uniform across all immunotherapy-based strategies. The most convincing data currently concern PD-1 inhibitor-based combinations in recurrent or metastatic disease, whereas the roles of immunotherapy in locally advanced disease, in combination with radiotherapy, in the adjuvant setting, or in rechallenge strategies remain less clearly established [62,64,93,94,95,96,97,98,99]. This creates an important distinction between approaches already supported by phase III evidence and those that are still biologically attractive but insufficiently validated for routine clinical decision-making [93,94,95,96,97,98].

From a clinical decision-making perspective, current evidence supports a stage-adapted approach to nasopharyngeal carcinoma management. In locoregionally advanced nasopharyngeal carcinoma, intensity-modulated radiotherapy combined with concurrent platinum-based chemotherapy remains the therapeutic backbone, while induction chemotherapy may be considered in patients with higher-risk features, particularly those at increased risk of distant failure [7,51,52,53,54,55,56,57,58,59,66,67,68,69,70,71,72,73,74]. The addition of immune checkpoint inhibitors in this setting is promising, especially in high-risk locoregionally advanced disease, but the optimal treatment sequence, target population, and long-term survival benefit still require further validation [65,145,146,147]. In contrast, in recurrent or metastatic nasopharyngeal carcinoma, the evidence for immunotherapy is more mature. Phase III trials, including JUPITER-02, CAPTAIN-1st, and RATIONALE-309, support the use of programmed death-1 inhibitor-based combinations with chemotherapy as a preferred first-line systemic strategy in this clinical setting [93,94,95,96,97,98,148,149]. Biomarkers may further refine treatment selection, although their clinical maturity differs substantially. Circulating Epstein–Barr virus DNA is currently the most clinically useful marker for risk stratification, response monitoring, recurrence detection, and potentially treatment de-escalation in selected patients [108,109,110,111,112,113,114,115,116,117,118]. By contrast, programmed death-ligand 1 expression, tumor immune microenvironment features, tertiary lymphoid structures, transcriptomic signatures, liquid biopsy markers, and radiomic models remain promising but insufficiently standardized for routine treatment selection [97,100,101,119,120,121,122,123,124,125,126,127,128,129,130,131,132,133,134,135,136,137,138,139,140,147]. Therefore, emerging therapies and biomarkers should currently be interpreted as tools supporting treatment stratification rather than as universal determinants of therapy choice.

The role of PD-L1 as a biomarker also requires particularly cautious interpretation. Although PD-L1 expression is biologically plausible in NPC and closely linked to the EBV-driven and immunologically active tumor microenvironment, its predictive and prognostic value remains inconsistent across studies [59,60,61,62,97,102,103]. This inconsistency likely reflects methodological heterogeneity, including differences in assay platforms, scoring systems, cutoff values, treatment settings, and the use of monotherapy versus combination regimens [97]. Therefore, PD-L1 should currently be viewed as an incomplete and non-standalone biomarker in NPC, whereas circulating EBV DNA appears to be more clinically mature for risk stratification and disease monitoring [97,108,109,110,111,112,113,114,115,116,117,118].

In contrast, targeted therapy currently appears to be a less promising direction. Although the EGFR and VEGF pathways are among the most frequently investigated potential therapeutic targets, their clinical value in NPC has not yet been unequivocally confirmed [82,83,84,85]. This illustrates that even with an increasingly detailed understanding of tumor molecular biology, translating biological observations into effective and widely applicable targeted therapies remains challenging. On the other hand, this very biological complexity further reinforces the importance of precision medicine. The most clinically mature biomarker remains circulating EBV DNA, which may have applications in diagnosis, monitoring treatment response, detecting recurrence, and perhaps also in de-escalation strategies for selected patients [108,109,110,111,112,113,114,115,116,117,118,119,120,121,122,123,124]. At the same time, advances in genomic, transcriptomic, and liquid biopsy analyses suggest that the future of NPC management will increasingly rely on the integration of multiple biomarker classes rather than on individual indicators alone [109,110,111,112,113,114,115,116,117,118,119,120,121,122,123,124,125,126,127,128,129,130,131]. This illustrates a broader challenge in NPC research: the rapid expansion of molecular knowledge has not yet translated into a comparable number of clinically actionable targets [82,83,84,85,86,87,88,89,90]. In many cases, the literature identifies biologically interesting pathways or candidate biomarkers, but prospective biomarker-driven validation remains limited [82,83,84,85,86,87,88,89,90,119,120,121,122,123,124,125,126,127,128,129,130,131]. As a result, precision medicine in NPC is advancing conceptually faster than it is being implemented in everyday clinical practice [119,120,121,122,123,124,125,126,127,128,129,130,131].

Particular attention should also be paid to the fact that recent studies increasingly describe NPC not only through the prism of classical clinical features, but also in terms of the composition and dynamics of the TME. Signatures related to TLS, the level of immune infiltration, T-cell exhaustion, and remodeling of macrophage populations may, in the future, help better predict response to immunotherapy [121,122,123,124]. Similarly, radiomics, models based on MRI, PET/CT, and artificial intelligence tools have the potential to improve prognostication, diagnosis, and radiotherapy planning [132,133,134,135,136,137,138,139,140]. However, caution in interpretation is warranted, as the currently available data are characterized by substantial methodological heterogeneity, limited standardization, and a risk of publication bias [134,135,136,137,138,139,140]. This caution is especially important because many studies in radiomics, artificial intelligence, and transcriptomic stratification are retrospective, single-center, and based on relatively small or highly selected cohorts [121,122,123,124,134,135,136,137,138,139,140]. In addition, external validation remains limited, and model reproducibility across institutions has not yet been adequately demonstrated [134,135,136,137,138,139,140]. Consequently, these tools should currently be regarded as promising research instruments rather than fully established clinical decision-support systems [134,135,136,137,138,139,140]. This means that both molecular biomarkers and advanced imaging biomarkers are highly promising, but for the most part still remain in transition from translational research to full clinical implementation.

Although surgery was outside the primary scope of this review, recent studies on the salvage treatment of recurrent NPC further illustrate several broader challenges in the field [5,150]. A retrospective study from a non-endemic center reported 5-year overall, disease-specific, and disease-free survival rates of 60.7%, 69.0%, and 39.7%, respectively, after salvage endoscopic nasopharyngectomy, with outcomes significantly influenced by recurrent stage and margin status [5]. In parallel, a recent systematic review with meta-analysis showed that positive surgical margins were associated with worse 5-year disease-free survival, while also highlighting the lack of uniform margin definitions across studies [150]. Taken together, these findings reinforce the need for better standardization, more consistent reporting, and clearer patient selection criteria across NPC treatment research.

In summary, current evidence suggests that the future of NPC treatment will not lie in replacing existing standards, but rather in integrating them more effectively. IMRT and CCRT remain the foundation of treatment for locally advanced disease; however, further improvement in outcomes will most likely depend on more precise selection of systemic therapy, better integration of immunotherapy, and implementation of reliable biomarkers for patient stratification [7,8,9,10,11,12,51,52,53,54,55,56,57,91,92,93,94,95,96,108,109,110,111,112,113,114,115,116,117,118,119,120,121,122,123,124,125,126,127,128,129,130,131,132,133,134,135,136,137,138,139,140]. Therefore, the most important challenges for the coming years include not only the development of new treatment modalities, but also the generation of stronger comparative evidence, prospective validation of predictive biomarkers, standardization of biomarker assessment, and clearer identification of those patient subgroups most likely to benefit from specific treatment strategies [7,8,9,10,11,12,51,52,53,54,55,56,57,91,92,93,94,95,96,108,109,110,111,112,113,114,115,116,117,118,119,120,121,122,123,124,125,126,127,128,129,130,131,132,133,134,135,136,137,138,139,140].

## 9. Conclusions

Despite recent treatment advances, NPC remains a therapeutic challenge requiring a holistic approach. NPC treatment visually shifts towards a multimodal, personalized approach, combining traditional treatment methods with modern systemic strategies. Significant progress has been made in radiotherapy, especially IMRT, and its combination with chemotherapy. This multidrug treatment approach led to better disease control and patients’ OS and PFS. However, metastases remain the main reason for disease-related deaths and treatment failure, highlighting the need for further systemic therapy developments.

Immunotherapy is of particular importance as it addresses the biological distinctive features of NPC better than therapies targeting single molecular pathways. Immunotherapy, mainly ICIs PD-1 and PD-L1, which, when combined with chemotherapy, have shown promising results. Advances in precision medicine, including biomarkers such as EBV DNA, may play a crucial role in NPC early diagnosis, treatment outcomes prediction, and personalized therapy. Moreover, molecular analysis and radiomics may improve clinical decision-making in the future.

Moreover, future advancements in NPC treatment outcomes will result from optimal integration and alignment of the treatments with the disease’s biology. In clinical practice, specialists will take into account molecular and immunological factors such as EBV DNA levels, TME characteristics, and gene expressions.

A futuristic approach will also cover reliable and standardized predictive biomarkers, implementing the results in daily medical practice. That being said, integrating the development of liquid biopsy and AI-based tools may allow for a more personalized treatment of NPC.

Summing up, the future of NPC treatments will focus on integrating present treatments and tailoring therapies to each patient. Further perfecting of systemic treatment strategies, the identification of reliable predictive biomarkers, and the optimal selection of therapies for specific patient groups will be of key importance.

## Data Availability

Not applicable.

## Abbreviations

ACT	Adoptive Cell Transfer
AJCC/UICC	American Joint Committee on Cancer/International Union Against Cancer
ART	Adaptive Radiotherapy
CCRT	Concurrent Chemioradiotherapy
cfDNA	Cell-free DNA
CNV	Copy Number Variation
ctDNA	Circulating Tumor DNA
DFS	Disease-Free Survival
DMFS	Distant Metastasis-Free Survival
EBV	Epstein–Barr Virus
GP	Platinum-Gemcitabine
HLA	Human Leukocyte Antigen
HNSCC	Head and Neck Squamous Cell Carcinoma
ICB	Immune Checkpoint Blockade
ICIs	Immune Checkpoint Inhibitors
ICT	Induction Chemotherapy
IMRT	Intensity-Modulated Radiotherapy
K-NPC	Keratinizing Nasopharyngeal Carcinoma
LANPC	Locoregionally Advanced Nasopharyngeal Carcinoma
mid-EBV	EBV DNA Detected at Week 5 of Radiotherapy
MRD	Minimal Residual Disease
NK-NPC	Nonkeratinizing Nasopharyngeal Carcinoma
NPC	Nasopharyngeal Carcinoma
OS	Overall Survival
PD-1	Programmed Death-1
pEBV	Plasma Epstein–Barr Virus
PFS	Progression-Free Survival
post-EBV	Detectable EBV DNA After Treatment
PRT	Proton Therapy
RNA-seq	RNA Sequencing
SCC	Squamous Cell Carcinoma
TAMs	Tumor-Associated Macrophages
TIME	Tumor Immune Microenvironment
TLS	Tertiary Lymphoid Structures
TME	Tumor Microenvironment
TPF	Docetaxel, Cisplatin, 5-Fluorouracil
